# Converging Prefronto-Insula-Amygdala Pathways in Negative Emotion Regulation in Marmoset Monkeys

**DOI:** 10.1016/j.biopsych.2017.06.016

**Published:** 2017-12-15

**Authors:** Yoshiro Shiba, Lydia Oikonomidis, Stephen Sawiak, Tim D. Fryer, Young T. Hong, Gemma Cockcroft, Andrea M. Santangelo, Angela C. Roberts

**Affiliations:** a Department of Physiology, Development and Neuroscience, Wolfson Brain Imaging Centre, University of Cambridge, Cambridge, United Kingdom; b Department of Psychology, Wolfson Brain Imaging Centre, University of Cambridge, Cambridge, United Kingdom; c Behavioural and Clinical Neuroscience Institute, Wolfson Brain Imaging Centre, University of Cambridge, Cambridge, United Kingdom; d Department of Clinical Neurosciences, Wolfson Brain Imaging Centre, University of Cambridge, Cambridge, United Kingdom

**Keywords:** Amygdala, Emotion regulation, Insula, Orbitofrontal cortex, Prefrontal cortex, Ventrolateral prefrontal cortex

## Abstract

**Background:**

Impaired regulation of emotional responses to potential threat is a core feature of affective disorders. However, while the subcortical circuitry responsible for processing and expression of fear has been well characterized, the top-down control of this circuitry is less well understood. Our recent studies demonstrated that heightened emotionality, as measured both physiologically and behaviorally, during conditioned fear and innate/social threat was induced, independently, by excitotoxic lesions of either the anterior orbitofrontal cortex (antOFC) or ventrolateral prefrontal cortex (vlPFC). An important outstanding question is whether the antOFC and vlPFC act on common or distinct downstream targets to regulate negative emotion.

**Methods:**

The question was addressed by combining localized excitotoxic lesions in the PFC of a nonhuman primate and functional neuroimaging ([^18^F]fluorodeoxyglucose positron emission tomography) with a fear-regulating extinction paradigm. Marmoset monkeys with unilateral lesions of either the antOFC or vlPFC were scanned immediately following exposure to a fearful or safe context, and differences in [^18^F]fluorodeoxyglucose uptake were evaluated.

**Results:**

[^18^F]fluorodeoxyglucose uptake in the insula and amygdala of the intact hemisphere was significantly increased in response to the fearful context compared with the safe context. Such discrimination between the two contexts was not reflected in the activity of the insula-amygdala of the antOFC or vlPFC-lesioned hemisphere. Instead, uptake was at an intermediate level in both contexts.

**Conclusions:**

These findings demonstrate that the distinct control functions of the antOFC and vlPFC converge on the same downstream targets to promote emotion regulation, taking us closer to a mechanistic understanding of different forms of anxiety.

Mood and anxiety disorders cause significant disturbance in the everyday life of those affected and are a serious burden on families and society at large (1). One of the hurdles against the development of efficacious treatment is the fact that individual patients show marked variation in treatment response, with some not responding at all, despite having the same diagnosis (2). This suggests that although the observable symptoms appear to be similar, the underlying neurobiological mechanisms may differ between individuals. Therefore, to refine current interventions and develop more efficacious treatments, it is essential that we identify the distinct neural circuits that may underlie these symptoms of unregulated and excessive anxiety.

Converging evidence from correlative neuroimaging 3, 4 and neurosurgical studies in humans (5) and experimental lesion studies of fear conditioning and extinction in rodents (6) highlight the importance of the ventromedial prefrontal cortex (vmPFC) and its top-down inhibition of the amygdala and/or insula cortex in the control of negative emotion. Although other prefrontal areas have been implicated in the regulation of negative emotion in humans 7, 8, whether they act through the same downstream circuits is less clear. This is due primarily to the paucity of studies investigating the effects of selective manipulations of these other prefrontal brain regions in animals in which their causal contribution can be determined. Recently, our laboratory compared the effects of selective fiber-sparing excitotoxic lesions of either the anterior orbitofrontal cortex (antOFC) or ventrolateral PFC (vlPFC) of marmoset monkeys on both physiological and behavioral responses during exposure to threatening stimuli. These prefrontal brain regions in the marmoset (9) show a very similar cytoarchitectonic parcellation to that seen in humans and rhesus macaques (10), making findings highly translatable into the clinical setting. Three different tests simulated three different anxiety-provoking conditions: learning to predict a threat (pavlovian discriminative fear conditioning), dealing with an unfamiliar social stimulus (human intruder test), and responding to an innately fearful object (rubber snake test). In all three conditions, lesions of either the vlPFC or antOFC induced stronger, less adaptable cardiovascular and behavioral responses 11, 12 that were relatively indiscriminate from one another. Only when their effects were compared on an approach–avoidance decision-making task were their individual contributions differentiated. The vlPFC was implicated in online attentional control of emotional responses, while the antOFC modulated the subsequent establishment of punishment memories (13). An outstanding and important question is whether the antOFC and vlPFC are part of a common neural network for regulating negative emotion, acting on the same downstream pathways involved in the expression of emotion.

Here we addressed this empirical gap by combining localized excitotoxic lesions of either the antOFC or vlPFC and [^18^F]fluorodeoxyglucose ([^18^F]FDG) positron emission tomography (PET) with a fear-inducing behavioral paradigm. Functional neuroimaging enabled visualization and comparison of the effect of the lesion on activity in downstream structures during threat exposure (i.e., a rubber snake) and following extinction (acquired safety). Because the antOFC and vlPFC send very few projections to extraprefrontal targets in the contralateral hemisphere 14, 15, a unilateral lesion model enabled animals to act as their own control subjects. An A-B-A behavioral design, in which the safety condition was sandwiched between two fear conditions, provided scan replicability within animals.

## Methods and Materials

All procedures were conducted in accordance with the U.K. 1986 Animals (Scientific Procedure) Act under project license PPL70/7618.

### Subjects

**Figure 1:**
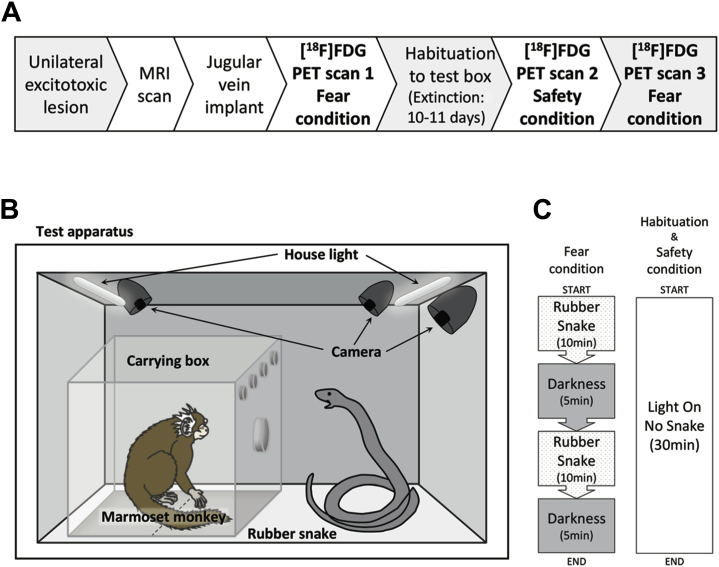
**(A)** Order of the experimental procedures. **(B)** Schematic diagram of the test apparatus. Light was provided by two LED light tubes, lined along the ceiling edges. Three cameras mounted on the inside walls of the chamber recorded the behavior of the subject. The dotted line on the floor of the carrying box represents the imaginary line between the zones near to and far from the snake. The model snake resembled a coiled cobra with its head raised (27 cm in height) and was dark brownish in color with black stripes. **(C)** Sequence of stimulus presentations for the fear and safety conditions. [^18^F]FDG, [^18^F]fluorodeoxyglucose; MRI, magnetic resonance imaging; PET, positron emission tomography.

A total of 14 adult common marmosets (*Callithrix jacchus*; average age 3.2 ± 1.0 years) were used. Of these marmosets, 7 (3 female and 4 male) received unilateral antOFC excitotoxic lesions, and 4 (2 female and 2 male) received unilateral vlPFC excitotoxic lesions 14 ± 1 weeks before scanning. To assess the physiological impact of the rubber snake presentation, an additional 3 marmosets (all male) received a wireless telemetry probe inserted into the descending aorta in a single surgical operation and received the same series of behavioral tests as the lesioned subjects described below but without being scanned. See Supplemental Methods and Materials for further details, including housing and diet information. The order of experimental procedures is described in Figure 1A.

### Excitotoxic Lesion Surgery

**Figure 2:**
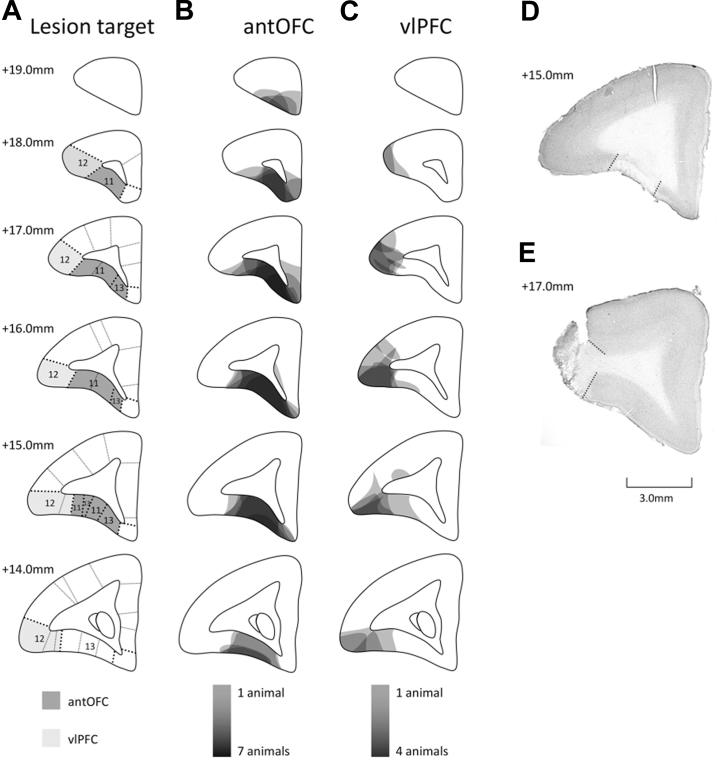
**(A–C)** Schematics of coronal sections through the frontal lobe (anterior–posterior) of the left hemisphere of the marmoset brain. **(A)** The dark and pale shading represents the anterior orbitofrontal cortex (antOFC) and ventrolateral prefrontal cortex (vlPFC) areas targeted for the excitotoxic lesion, respectively. The numbers indicate anterior–posterior coordinates from the interaural line. Numerical designations reflect cytoarchitechtonic regions identified within the PFC, redrawn from Burman and Rosa (44). **(B, C)** The pale to dark shading reflects the area of cell loss in 1 to 7 animals with excitotoxic lesions of the antOFC **(B)** and 1 to 4 animals with excitotoxic lesions of the vlPFC **(C)**. For illustrative purposes, those in which lesions were placed in the right hemisphere are shown on the left. All animals in the antOFC-lesioned group sustained significant neuronal cell loss throughout area 11 and anteromedial area 13. In the vlPFC-lesioned group, all animals sustained marked neuronal loss throughout the anterior sector of area 12, with more varied neuronal loss in more caudal sectors. **(D, E)** Photomicrographs of a representative coronal section through the PFC showing the lesion extent (dotted line) for the antOFC **(D)** and vlPFC **(E)**.

Surgical procedures were described in detail in an earlier report (10); see Supplemental Methods and Materials for details. Figure 2A illustrates the lesion targets. A unilateral lesion model was used so that animals could act as their own control subjects, reducing experimental variation caused by interanimal differences in [^18^F]FDG uptake. In contrast to extensive projections from the PFC to ipsilateral regions, very few projections cross the midline and project directly to extraprefrontal regions in the contralateral hemisphere 14, 15.

### Port Implant Surgery

A vascular access port (Solomon Scientific, Skokie, IL) was implanted in the animal to allow swift subcutaneous injection of [^18^F]FDG, reducing the animal’s discomfort and the experimenter's exposure to radioactivity. Details of surgical procedures are described in Supplemental Methods and Materials.

### Cardiovascular Implant Surgery

A wireless telemetry probe was inserted into the descending aorta of 3 marmosets to allow the remote measurement of their cardiovascular activity while freely moving. The surgical procedure was the same as described previously (16) and is reported in Supplemental Methods and Materials.

### Magnetic Resonance and Positron Emission Tomography Imaging

#### Magnetic Resonance Imaging Scan

Between the lesion surgery and port implant, the animals received a magnetic resonance imaging scan under isoflurane anesthesia using a rapid acquisition with relaxation enhancement sequence at 4.7T with a Bruker PharmaScan 47/16 system (Ettlingen, Germany). For details, see Supplemental Methods and Materials.

#### PET Scan

Each animal received three [^18^F]FDG PET scans with a microPET Focus-220 scanner (Concorde Microsystems, Knoxville, TN), with the first scan occurring 14 ± 1 weeks after lesion surgery. For details of scan procedure and data analysis, see Supplemental Methods and Materials.

### Voxelwise Analysis of PET

SPM8 (Wellcome Trust Institute for Neurology, University College London) was used for voxel-based analysis. A general linear model was configured with covariates for subject and condition (fear vs. safety), and increased activity was tested with Student’s *t* test at each voxel. To control for type I errors anticipated as a result of multiple comparisons, the familywise error was controlled at *p* < .05 on a cluster level with a cluster-forming threshold of *p* < .001 (17).

### Behavioral Paradigm

#### Fear Induction

Immediately after the [^18^F]FDG injection, the animal was transported to a sound-attenuated test apparatus in a clear cuboidal Perspex box (Perspex Distribution Ltd, Weybridge, UK). The entire carrying box was fitted into the internal frame of the test apparatus (Figure 1B). A rubber snake was used as a fearful stimulus. It was placed inside the test apparatus next to the Perspex box. Previous studies showed that the snake induces anxiety-related behaviors in marmoset monkeys 12, 18, 19. The 30-minute test session was divided into four phases (Figure 1C). During the initial 10 minutes, the light in the box was on and the snake was visible. The light was then turned off for 5 minutes during which the inside of the box was completely dark. This 10-minute light and 5-minute dark sequence was repeated a second time. At the end of the 30-minute session, the animal was removed from the test apparatus and received the PET scan procedure described above.

#### Habituation

Habituation sessions started the day after the PET scan. Following the same procedure as above but without the [^18^F]FDG injection or scan, the animal was placed in the test apparatus for 30 minutes. The house light was kept on, and no snake was presented (Figure 1C). Following session completion, the animal was taken out and returned to the home cage. Each animal received 10 or 11 sessions until it appeared to be habituated to the apparatus, as indicated by a relaxed posture in the test box (20).

#### Safety

The day after the last habituation session, the animal received a second PET scan. Immediately after the [^18^F]FDG injection, the animal was placed in the same test apparatus as the previous habituation sessions (light on with no snake) for 30 minutes and then received the PET scan as before.

#### Fear Induction Replication

Two weeks after the safety condition, the animal went through the same procedure as the initial fear induction test using the same snake except in a different test apparatus within a different room.

An additional 3 animals that had received cardiovascular wireless implants but no brain surgery went through this same behavioral procedure without scanning. Cardiovascular measurements provided additional insight into the emotional responsiveness of animals to fear induction.

### Behavioral Analysis

To assess the impact of the rubber snake on the animal’s behavior (see Supplemental Methods and Materials for detailed description), each session was video-recorded, and the positioning of the marmoset in relation to the snake location was scored by a person blinded to the experimental groups using a quantitative analysis program (JWatcher, version 1.01; www.jwatcher.ucla.edu). The test box floor was divided into two major sections: near to and far from the snake (Figure 1B). Percentage of time spent in each of the sections was calculated and compared across different conditions.

### Cardiovascular Analysis

Blood pressure (BP) data transmitted by an implanted telemetry probe were analyzed following the procedure described in (11). For details, see Supplemental Methods and Materials.

### Histological Analysis

Details of euthanasia, histological preparation, and verification of lesions were described in (10) and are reported in Supplemental Methods and Materials.

### Statistical Analysis

As is often the case in experimental studies with animals, especially primates, there are concerns with type I errors when the sample sizes are relatively low. To mitigate this possibility, we used a powerful A-B-A design coupled with unilateral lesions such that the intact hemispheres of the same brains were used as controls. The statistics used were appropriately controlled for type I errors due to multiple comparisons, and the imaging results were confirmed with nonparametric bootstrap resampling.

SPSS (version 23; IBM Corp., Armonk, NY) was used to carry out statistical analyses. Because there was no significant difference in either behavioral or imaging data between the two fear-inducing sessions (Supplemental Figures S2 and S3), the data from the two sessions were averaged and this average score was used for subsequent statistical analyses. There was no significant difference in either behavior or [^18^F]FDG uptake between the left- and right-lesioned animals (Supplemental Figure S1A, B) and no effect of sex (Supplemental Results).

## Results

### The Insula-Amygdala Region in the Intact Hemisphere Shows Greater [^18^F]FDG Uptake in a Fear-Inducing Condition Compared With a Safety Condition

**Figure 3:**
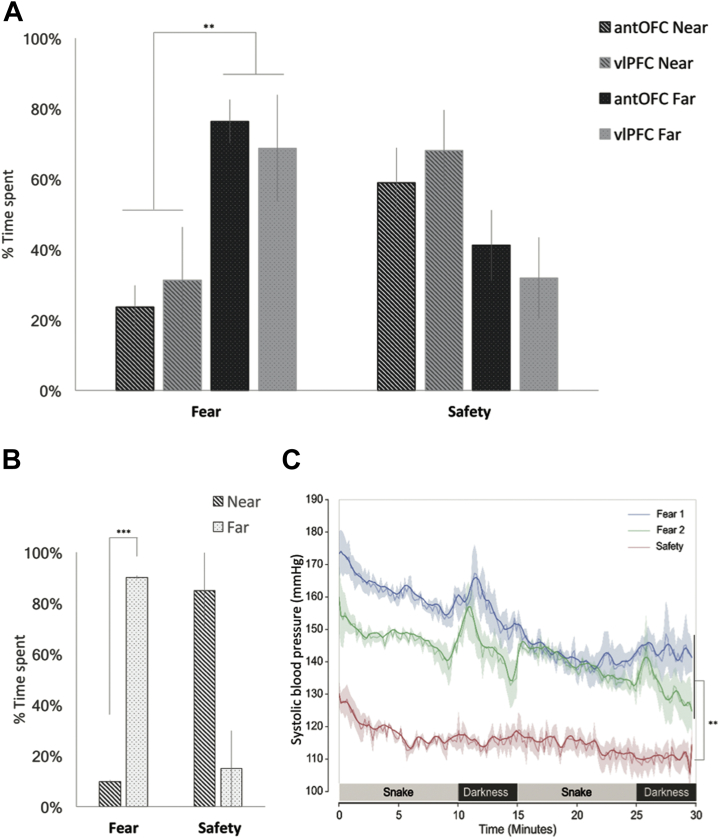
**(A)** Proportion of time spent in either the near (striped bar) or far (dotted bar) sector of the test box during the fear induction and safety conditions for the anterior orbitofrontal cortex (antOFC)-lesioned (black background bar) and ventrolateral prefrontal cortex (vlPFC)-lesioned (gray background bar) groups. **(B)** The same pattern was present in the 3 telemetry-implanted animals. Error bars indicate SE. **(C)** Accompanying systolic blood pressure (BP) is shown for these additional 3 animals, with increases occurring during the fear sessions compared with safety. Mean systolic BP (in millimeters of mercury [mmHg]) is plotted for the total duration (30 minutes) of the first fear session (blue), the safety session (red), and the second fear session (green). The data are presented after being smoothed using a Savitzky–Golay filter, which increases the signal-to-noise ratio for ease of visualization (bold line). The raw systolic BP trace is also presented (faint line). Shading represents ± SEM. The significance indicates the difference of the BP between the safety and the mean of two fear conditions for each phase: **p < .01, ***p < .001. Additional analyses (Supplemental Results) revealed that while there was a small but significant decline between the first and second fear conditions, importantly, both conditions were independently significantly different from the safety condition.

Exposure to a rubber snake and darkness produced a significant change in the animals’ behavior such that they remained as far away as possible from the corner in which the snake was placed. This is reflected in significantly greater time spent in the far sector compared with the near sector of the test box floor across both fear sessions. This behavior extinguished on repeated habituation to the same test apparatus in the absence of snake and darkness (extinction) (Figure 3A) [three-way analysis of variance (ANOVA): condition (fear vs. safety) × distance (near vs. far) × group (antOFC vs. vlPFC); condition × distance interaction, *F*_1,9_ = 10.926, *p* = 0.009; post hoc pairwise comparison revealed a significant difference in distance during the fear condition, *F*_1,9_ = 10.697, *p* = .010, but not during the safety condition, *F*_1,9_ = 2.848, *p* = .126]. In the 3 animals that received cardiovascular implants but were not scanned and had not received unilateral excitotoxic lesions, the same pattern of behavior was seen in the fear and safety conditions (two-way ANOVA: condition × distance interaction, *F*_1,2_ = 27.376, *p* = .035; post hoc pairwise comparison revealed a significant difference in distance during the fear condition, *F*_1,2_ = 4145.753, *p* < .001, but not during the safety condition, *F*_1,2_ = 5.555, *p* = .143) (Figure 3B and Supplemental Results). The similarity of the behavior of these 3 animals with that of the lesioned groups indicates that the unilateral lesions per se did not induce any marked change in behavior in the fear-inducing context compared with unoperated control subjects. The behavior in the unoperated control subjects was accompanied by marked increases in systolic BP during the fear condition compared with the safety condition (Figure 3C and Supplemental Results). Close examination of the cardiovascular activity during the fear condition suggests that the period of darkness inserted between the snake presentations enhanced the BP even further, consistent with the fact that marmosets, like humans, are a diurnal primate (21) and show a similar fear of darkness (22).

**Figure 4:**
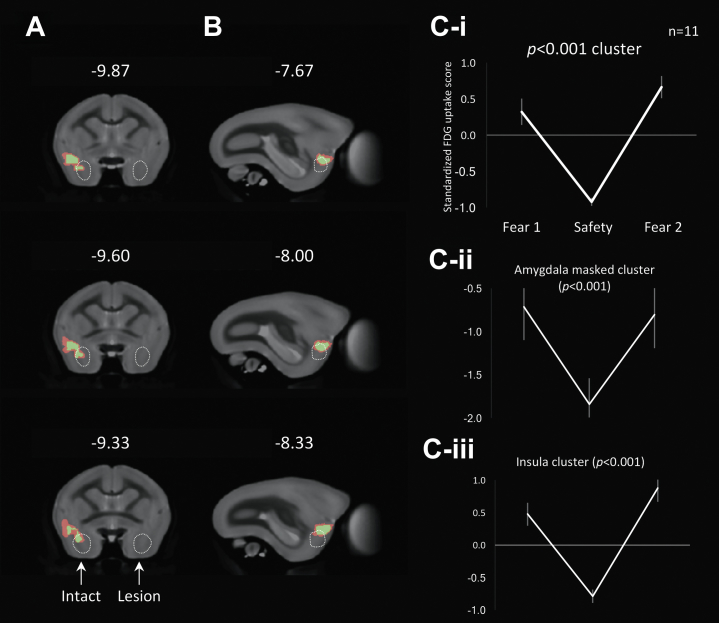
**(A, B)** Differences in fluorodeoxyglucose (FDG) uptake between fear and safety from the voxelwise analysis showing coronal sections (top–bottom: anterior–posterior) **(A)** and sagittal sections (top–bottom: medial–lateral) **(B)** from the intact side of the brain. For brain images with a left-sided lesion, the intact side is depicted on the left for illustrative purposes only. Numbers indicate the distance in millimeters from the interaural line for the coronal sections and the midline for the sagittal sections. Colored areas indicate regions with significantly greater fluorodeoxyglucose uptake (red: *p* < .005; green: *p* < .001) during the fear induction condition compared with the safety condition. The dotted white circle indicates the amygdala region of interest, based on the marmoset brain atlas (8). Line graphs show the mean of the standardized fluorodeoxyglucose uptake scores from the significant cluster (*p* < .001). **(C-i)** The same cluster masked by the amygdala region of interest **(C-ii)** and the remaining insula cluster **(C-iii)** across the three conditions. Standardized scores for the amygdala and remaining insula clusters were calculated using the mean and SD from the significant cluster. Error bars indicate SE.

Voxelwise analysis of the [^18^F]FDG uptake for the intact hemisphere revealed a cluster, centered on the insula and amygdala, showing significantly greater uptake during fear-inducing conditions (average scores of the first and second fear-inducing sessions) compared with the safety condition (Figure 4A, B; for visualization, a relaxed threshold of *p* < .005 is shown in red, while the stringent threshold cluster at *p* < .001 [used for all quantitative/statistical analyses] is shown in green). The line graph showing the mean of the standardized [^18^F]FDG uptake scores for the cluster (*p* < .001) reveals much greater uptake for both the first and second fear induction conditions compared with the safety condition (Figure 4C-i). The same pattern was observed when the [^18^F]FDG uptake scores for the area of the cluster masked with the amygdala region of interest (Figure 4C-ii) and for the remaining area encompassing the insula region (Figure 4C-iii) were compared across the three conditions. It can be seen that the dorsal sectors of the lateral and basal nuclei and the lateral sector of the central nucleus were the main areas of increased [^18^F]FDG uptake within the amygdala. The rest of the cluster fell within the agranular, dysgranular, proisocortex, and parainsular regions.

### antOFC and vlPFC Lesions Independently Attenuate the Differential [^18^F]FDG Uptake in the Insula-Amygdala Region to Fear and Safety

**Figure 5:**
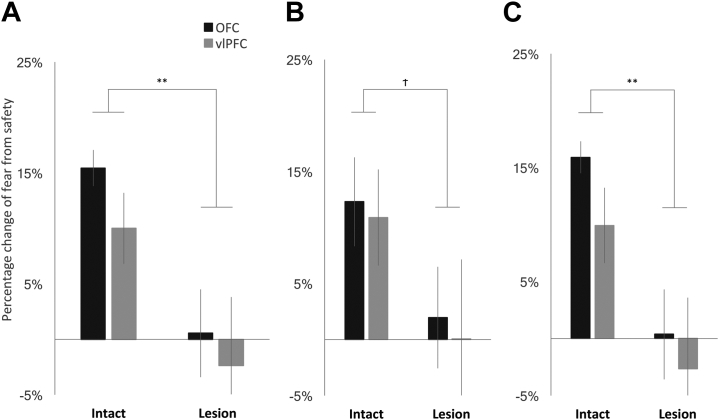
Graphs showing the fluorodeoxyglucose uptake in response to the fear-inducing condition (averaged across fear 1 and fear 2) proportional to the safety condition, for the *p* < .001 cluster identified by the voxel-based analysis **(A)**, the cluster masked with the amygdala region of interest **(B)**, and the remaining cluster encompassing the insula **(C)** for both intact and lesioned hemispheres. While the intact hemisphere exhibited a marked change between the fear-inducing and safety conditions, such a differential response was absent in the lesioned hemisphere. No significant difference between the anterior orbitofrontal cortex (OFC)-lesioned group (black bar) and the ventrolateral prefrontal cortex (vlPFC)-lesioned group (gray bar) was found. Error bars indicate SE. **p < .01, †*p* = .058.

Excitotoxin infused into the antOFC resulted in extensive cell loss throughout all subdivisions of area 11 and the anterior sector of area 13 in the lesioned hemisphere of all 7 animals. There was minimal encroachment on the more lateral region of area 12, and in only 2 animals did damage extend into areas 14 and 10 anteriorly (Figure 2B, D). In the vlPFC-lesioned group, extensive cell loss was restricted to the mid region of area 12, sparing the most anterior sector and the more caudal sectors (Figure 2C, E). Cell loss in both regions was similar in location to that shown previously to induce anxiety in marmosets when occurring bilaterally 11, 12. To assess the effect of the lesion on the differential [^18^F]FDG uptake response to the fear-inducing condition compared with the safety condition observed in the intact hemisphere, the outline of the significant cluster (*p* < .001) in the intact hemisphere was redrawn on the lesioned hemisphere. The [^18^F]FDG uptake values from each voxel within this cluster were then averaged. To quantify the proportional difference in the [^18^F]FDG uptake between the fear-inducing and safety conditions, percentage change from the safety condition to the fear-inducing condition was calculated. The analysis revealed that the differential response in the fear-inducing condition compared with the safety condition observed in the intact hemisphere was absent in the lesioned hemisphere for both the antOFC-lesioned and vlPFC-lesioned groups (Figure 5A) (two-way ANOVA: main effect of hemisphere [intact vs. lesion], *F*_1,9_ = 12.501, *p* = .006). No significant difference between lesioned groups or a group × hemisphere interaction was observed [group (antOFC vs. vlPFC), *F*_1,9_ = 1.197, *p* = .302; group × hemisphere, *F*_1,9_ < 1]. A similar pattern was found for the cluster masked with the amygdala region of interest (Figure 5B) (two-way ANOVA: trend level effect of hemisphere [intact vs. lesion], *F*_1,9_ = 4.697, *p* = .058; group [antOFC vs. vlPFC], *F*_1,9_ < 1; group × hemisphere, *F*_1,9_ < 1) and for the remaining area encompassing the insula region (Figure 5C) (two-way ANOVA: main effect of hemisphere, *F*_1,9_ = 13.467, *p* = .005; group [antOFC vs. vlPFC], *F*_1,9_ = 1.401, *p* = .267; group × hemisphere, *F*_1,9_ < 1). An additional bootstrap method confirmed the robustness of these findings (Supplemental Results and Supplemental Figure S4). The absence of the differential responses in the lesioned hemisphere in the fear versus safety conditions was due to the presence of intermediate levels in both conditions (Supplemental Figure S3).

## Discussion

Dedicated small-animal PET imaging of [^18^F]FDG was used to compare the effects of selective unilateral lesions of the antOFC and vlPFC on the modulation of metabolic activity in the brain related to the regulation of negative emotion in marmoset monkeys. Emotion regulation was assessed by comparing [^18^F]FDG uptake and the accompanying behavior in a fear-inducing context, in the same context after a series of extinction sessions in which fear-inducing stimuli were no longer presented (safety condition), and then in a second fear-inducing context. Initial exposure to the fear-inducing context (i.e., rubber snake and darkness) resulted in animals’ avoiding the snake, maintaining as great a distance from the snake as possible while in the test box, and exhibiting a marked rise in BP. These behavioral and cardiovascular responses declined across the extinction period and had reached a nadir by the time of the subsequent safety session. They reappeared during the second fear-inducing context. These changes in response to fear and safety conditions were reflected in alterations of metabolic activity in a circumscribed area of the forebrain of the intact hemisphere, which included the anterior insula and dorsal sector of the amygdala. In contrast, comparison of [^18^F]FDG uptake between the safety and fear conditions in the same region in the lesioned hemisphere revealed a marked blunting of the differential response following neuronal loss in either the antOFC or vlPFC. While the insula-amygdala cluster in the intact hemisphere showed a reduction in [^18^F]FDG uptake following extinction to the context, the equivalent cluster in the lesioned hemisphere showed intermediate levels in both fear and safety conditions.

### Top-Down Regulation of Emotion

The use of [^18^F]FDG small-animal PET to investigate changes in functional activity across neural networks as a consequence of localized brain manipulations has been relatively underexploited in primate experimental studies to date [but see (23)]. Here we demonstrate altered metabolic activity in a cluster encompassing the insula and dorsal amygdala following localized excitotoxic lesions of either the antOFC or vlPFC in marmosets. The reduction in metabolic activity within the amygdala and insula in response to a series of fear extinction sessions is consistent with functional neuroimaging reports implicating both of these regions in fear acquisition and expression, respectively [for a review, see (24)]. These two regions have extensive reciprocal connections (25), and a functional neuroimaging meta-analysis revealed significant coactivation of the ventral anterior insula (part of the cluster in the current study) with the amygdala in relation to physiological processing in negative emotional states (26). Enhanced activity and generalization of activity in these two brain regions during fear conditioning and extinction is associated with both high trait anxiety (27) and anxiety disorders (24). However, this is the first study to show a causal link between localized lesions of either the antOFC or vlPFC and dysregulated metabolic activity in both the insula and amygdala during fear conditioning and extinction. The pattern of the deficit implicates these regions in regulating downstream activity in response to changes in the relationship between environmental stimuli and negative events. Specifically, whereas the metabolic activity in the amygdala and insula of the intact hemisphere differentiated between the fear and safety conditions, these same regions did not show such differentiation in the lesioned hemisphere. Because there appear to be few contralateral projections from the antOFC and vlPFC to target brain regions outside of the PFC (15), the most likely explanation of these effects is that the amygdala in the lesioned hemisphere was no longer under effective regulation by the antOFC or vlPFC in that hemisphere. It should be noted, however, that the behavioral response differentiated between the fear and safety conditions of these unilaterally lesioned monkeys, as reflected in their positioning in the test apparatus farthest away from the snake in the fear context only. This was similar in extent to the 3 unoperated control monkeys. Thus, a unilateral lesion of either the antOFC or vlPFC and the corresponding loss of differential activity to fear versus safety in the amygdala on the ipsilateral side did not appear to alter the regulation of their behavior between these two contexts. This is consistent with the lack of behavioral effects of large unilateral lesions of the primate OFC on reward-related decision making (28). In contrast, unilateral lesions of the amygdala can induce a partial blunting of conditioned freezing in rodents during fear conditioning (29), and a combination of large ablations of OFC and excitoxic lesions of the amygdala on one side only in rhesus monkeys disrupts snake fear but has no effect on responsivity to a human intruder (30). We did not implant cardiovascular probes into the lesioned animals in the current study for reasons of welfare given that the animals had already received excitotoxic lesions and a newly established procedure of a jugular port implant. Thus, whether the cardiovascular responsivity, as distinct from the animals’ behavioral responsivity, was affected by these unilateral lesions could not be determined.

There is considerable evidence demonstrating the importance of the vmPFC in regulating fear expression and related activity in the amygdala of rodents. In particular, the infralimbic cortex has been implicated in the downregulation of the amygdala during extinction of conditioned fear (31). This is supported by findings of altered activity in the vmPFC of humans [proposed homologue of infralimbic cortex, but see (32)] during the recall of extinction of a conditioned fear response (33). Indeed, activity in both the amygdala and insular cortex in human functional neuroimaging studies has been related inversely to activity in the vmPFC in trait anxious subjects and in the vmPFC and rostral and dorsal anterior cingulate cortices in those with anxiety disorders 34, 35. Causal evidence for such interactions between vmPFC and the insular cortex in humans was provided recently in a study of patients with vmPFC damage (5). However, the damage was extensive and included not only areas 10, 14, 25, and 32 on the medial wall but also areas 11 and 12 in the orbitofrontal cortex. Because altered structural and functional activities within many of these distinct prefrontal brain regions have been associated with disorders of emotion regulation, including anxiety (36), which of these regions, when damaged, was responsible for the observed effects on insular activity could not be evaluated.

The current results demonstrate that areas 11 (antOFC) and 12 (vlPFC) could independently contribute to these effects, separate from any influence from the more medial regions of areas 14, 25, and 32. We reported previously that excitotoxic lesions of either the vlPFC or antOFC in the marmoset enhance anxiety and innate fear and cause conditioned fear responses to become more rigid 11, 12. Importantly, the finding that their inactivation induced a differential pattern of impairment on performance of an approach–avoidance decision-making task demonstrated their independent functional contribution to emotion regulation (13) and led to the hypothesis that anxiety was enhanced by OFC inactivation due to uncertainty and by vlPFC inactivation due to attentional inflexibility (37). This is consistent with the prominent role of the OFC in tracking changing reward and punishment contingencies 38, 39, 40 and of the vlPFC in attentional control 8, 41. Both the antOFC and vlPFC have reciprocal connections with the amygdala in marmosets (15) and rhesus monkeys (42), and both send projections to the neighboring insula [rhesus monkeys (43) and marmosets (15)]. Thus, both are in a position to modify activity directly in the insula cortex and amygdala.

These findings have important implications for our understanding of the etiology and treatment of psychiatric disorders involving dysregulation of negative emotion. The majority of experimental studies in animals have so far focused on the vmPFC and its regulation of the amygdala in fear conditioning and extinction and disruption of these circuits in anxiety disorders such as phobias and posttraumatic stress disorder (6). However, dysregulation may also be present in other prefrontal areas in these disorders, including the vlPFC and antOFC (36). The findings from the current neuroimaging study demonstrate the critical involvement of these additional prefrontal brain regions in the regulation of negative emotion. Together, they highlight the presence of multiple, functionally distinct prefronto-limbic pathways involved in emotion regulation that converge on the same threat detection/response system consisting of interconnected limbic structures that include the insula and amygdala. Individual differences in activity in these distinct prefronto-limbic pathways are likely to contribute to the varied etiology and responsivity to treatment among patients with mood and anxiety disorders. Characterization of the cellular and molecular mechanisms underlying the regulation of the amygdala and insula by these independent prefrontal pathways may reveal novel targets for their differential modulation. Such an approach will bring us closer to the development of individually tailored treatment strategies for symptoms of anxiety and fear dysregulation.
