# Supplementary material for: Converging Prefronto-Insula-Amygdala Pathways in Negative Emotion Regulation in Marmoset Monkeys
Source: Biol Psychiatry. 2017 Dec 15;82(12):895–903. doi: 10.1016/j.biopsych.2017.06.016 (PMC5697497; doi:10.1016/j.biopsych.2017.06.016)
Supplement: Supplemental Material [file mmc1.pdf]

Converging Prefronto-Insula-Amygdala Pathways in Negative Emotion Regulation in Marmoset Monkeys

Supplemental Information

Supplemental Methods and Materials

Animals: Number, Housing and Diet

Seventeen adult common marmosets (*Callithrix jacchus*; 7 female, 10 male, average age 3.2±1.0 years) entered the study. Eight marmosets (4 female, 4 male) received unilateral antOFC excitotoxic lesions and six marmosets (3 female, 3 male) received unilateral vlPFC excitotoxic lesions. Due to a port implant blockage in one animal, the final number in the antOFC lesioned group was seven (3 female, 4 male) and two animals in the vlPFC lesioned group were removed from the study because their lesion was either too extensive, extending into the antOFC or too limited. Thus the final number of vlPFC lesioned animals was 4 (2 female, 2 male). Three additional animals (3 males) received cardiovascular implants.

The animals were housed in male/female pairs in rooms with controlled humidity and temperature and with a 12-h light/dark cycle. They were fed wholemeal bread, hard-boiled egg, and a piece of fruit on weekdays. This diet was supplemented with additional fruit and nuts on the weekends. Water was available *ad libitum*.

Telemetry Recording System

To measure blood pressure (BP) changes remotely in freely moving animals, a PhysioTel Telemetry System (Data Sciences, Inc. (DSI), St Paul, Minnesota, USA) was used. The system consisted of five basic components:

1) An implantable transmitter (TA11PA-C40, DSI) which continuously detected and transmitted BP from within the animal via radio-frequency signals;

2) A receiver (RPC-1, DSI) located underneath the behavioral testing box, which received the digitized information from the implanted transmitter and relayed the data for subsequent translation;

3) A calibrated pressure output adapter (R11CPA, DSI) with an ambient pressure reference monitor (APR-1, DSI) to convert the absolute pressure measured by the implanted transmitter into gauge pressure in millimeters of mercury (mmHg);

4) An analogue-digital converter (Micro 1401, Cambridge Electronic Design (CED), Cambridge, UK), which converted the digitized data into an analogue (continuous wave) signal; and

5) Data acquisition software (Spike2, Version 7.02, CED) for collection, analysis and storage of the accumulated data.

Excitotoxic Lesion Surgery

All surgeries were performed under aseptic conditions. The animals were premedicated with ketamine hydrochloride (sedative, 0.1ml of a 100 mg/ml solution, intramuscular (i.m.); Amersham Pharmacia and Upjohn, Piscataway, NJ, USA) and carprofen (prophylactic analgesic, 0.03ml, subcutaneous (s.c.)), and anesthetized by isoflurane intubation (flow rate 2–2.5%; IsoFlo, Abbott Laboratories, Abbott Park, IL, USA). The animals were placed into a stereotaxic frame (David Kopf, Tujunga, CA, USA) with their head securely fixed in position with specially modified incisor and zygoma bars. A standardization technique (1) was used to determine the appropriate injection sites for each animal independently, based on the thickness of the marmoset's frontal pole. Excitotoxic lesions of the antOFC and vlPFC were then made by infusing 0.4-1.6 µl/site of a 0.09M solution of quinolinic acid unilaterally into six sites (Figure 2 A). The hemisphere that was lesioned was counterbalanced (antOFC: 4 left, 4 right; vlPFC: 3 left, 3 right). For all placements, infusions were made at a rate of 0.1µl / 20s by using a 2-µl precision Hamilton sampling syringe (Precision Sampling, Baton Rouge, LA, USA) through a stainless-steel cannula (30 gauge). The cannula remained in place for 4min, after which it was slowly withdrawn from the brain. The skin was sutured and covered with a

protective barrier (Germoline New Skin; Bayer, Newbury, UK), and dexamethasone phosphate (0.2ml i.m.; Fauling Pharmaceuticals plc, Warwicks, UK) was administered to avoid the unlikely event of tissue inflammation. Non-steroidal analgesics (0.1ml Metacam oral; St. Joseph, MO, USA) were given for 3 days after surgery at 24-h intervals. The animals had at least a 2-week recovery period.

Port Implant Surgery

Immediately prior to the surgery, the animal was intubated and anesthetized, following the same procedure described above. The animal was placed on a surgical table in a prone position. A small incision was made below the shoulder blades at right angle to the axis of the spine on the animal's back where a soloport would be placed. Another small incision was made on the neck to expose the jugular vein. A catheter attached to the port was then threaded under the skin from the back towards the neck. The port was placed in the skin pocket on the back. Through a small cut made in the jugular vein, the open end of the catheter was inserted in the direction of the heart. The catheter was glued to the vein with Vetbond (M3 Animal Care Products, MN, USA) and the incisions on the back and neck sutured. Following the surgery, analgesics (0.1ml Metacam oral; St. Joseph, MO, USA) and antibiotics (0.25ml Synulox oral; Pfizer Ltd., Kent, UK) were given daily for 3 and 7 days respectively. The soloport was flushed with Hepsal post-surgery on days 1, 3, 6, 10, 15 and then weekly. The animals had at least 10 days recovery period before behavioral testing began.

Cardiovascular Implant Surgery

One day prior to surgery, animals received prophylactic antibiotic treatment: 0.25ml Flagyl-S (40mg/ml metronidazole; Winthrop Pharmaceuticals., Guildford, UK) and 0.25ml Synulox (50mg/ml clavlanate-potentiated amoxicillin; Pfizer Ltd., Kent, UK). For implantation of telemetry probes, marmosets were premedicated with ketamine hydrochloride (sedative, 0.1ml of a 100 mg/ml solution, intramuscular (i.m.); Amersham Pharmacia and Upjohn, Piscataway, NJ, USA) and carprofen (prophylactic analgesic, 0.03ml, subcutaneous (s.c.)),

and anesthetized by isoflurane intubation (flow rate 2–2.5%; IsoFlo, Abbott Laboratories, Abbott Park, IL, USA). The animal was placed in a supine position onto a sterile drape and the limbs were secured with masking tape to allow unrestricted access to the abdomen. Under aseptic conditions, a 4-6cm midline abdominal incision was made using scissors to allow a clear view of the aorta from the upper portion of the vessel down to the bifurcation of the aorta to the renal arteries. The aorta was then carefully dissected from the surrounding fat and connective tissue. Once isolated, the lower portion of the aorta, just above the bifurcation, was lifted and a cotton thread, approximately 8cm in length, was passed underneath. The two ends were then clamped together with forceps to 1) lift the vessel for implantation and 2) to exert a small amount of tension to prevent blood reflux after blood flow to the area was restricted during catheterisation. Once the vessel was clear, to restrict blood flow, a finger was used to apply pressure to the upper most portion of the aorta and slight tension was placed on the thread at the base. Using a 23-gauge needle (bent at 60°, bevelled edge upwards), the vessel was punctured just above the bifurcation and the tip of the catheter was inserted using a catheter introducer. The catheter was then passed up the length of the vessel until approximately 30-40mm of the tubing was contained within the vessel. Once correctly positioned, the area was thoroughly dried and Vetbond (M3 Animal Care Products, Minnesota, USA) tissue adhesive was applied to the puncture site. After integrity of the seal was established, a cellulose patch was placed over the entry site and fixed in position with additional adhesive. Following implantation, the thread and retractors were removed and the abdominal cavity was moistened with sterile saline. The device body was then secured in position by incorporating the tabs on the implant into the muscle wall by using non-absorbable sutures (Ethilon 3-0 W; Ethicon Inc., Georgia, USA). After the closure of the muscle wall, the skin was closed using absorbable sutures (3-0 Vicryl W9444; Ethicon Inc., Georgia, USA) and Vetbond was applied to each stitch to ensure that the abdomen was completely sealed. Postoperative analgesia was maintained for three days with 0.1ml Metacam (1.5mg/ml meloxicam; Boehringer Ingelheim Vetmedica, Ingelheim/Rhein, Germany) given orally. Antibiotics, 0.25ml each of Flagyl and Synulox, were also administered orally for 10 days post-

surgery to protect against intestinal infection. Marmosets had a two-week recovery period before testing began.

MR and PET Imaging

MRI – An in-house custom-built quadrature birdcage coil was used for signal transmission and reception. Images were acquired with a matrix of 256 × 200 over a field of view of 6.40cm × 5.00cm yielding in plane resolution 250μm with 125 slices of 250μm. The repetition time was 11.75s with effective echo time of 23.5ms. Three repetitions were acquired at a bandwidth of 34.7kHz and averaged for a total scan time of 21min 44s.

PET scan – On the day of scan, the animals received no breakfast in order to lower blood glucose concentration and hence increase the transport of FDG into brain tissue, thereby increasing the cerebral FDG signal and hence reducing statistical noise in the PET image. Animals were placed in a test box approximately 3 minutes after a bolus injection of 71±14 MBq of FDG subcutaneously through the solo port. After 30 min of the behavioral paradigm described below, the animal was immediately intubated and anaesthetized following the procedure described above. The animal was then placed on the heatpad on the scanner bed and attached to monitoring equipment. Heart rate, SpO_2 and respirations were monitored continuously. The bed of the scanner was then positioned to locate the brain in the center of the PET scanner field of view, where both sensitivity and resolution are optimal. For consistency, PET data acquisition started 70min after the FDG injection and lasted for 45min. The energy and coincidence timing windows used were 350-650 keV and 6 nsecs, respectively.

The list mode data were histogrammed into 9 × 5min 4D sinograms, and then reconstructed using Fourier re-binning (FORE; (2)) followed by the 2D ordered subsets expectation maximization (OSEM; (3)) algorithm installed on the scanner (6 iterations, 16 subsets). As post-injection transmission scanning was not feasible, attenuation correction used a mean non-attenuation corrected FDG image to determine a body outline, within which

a uniform attenuation coefficient (0.096 cm^{-1}) was ascribed. This was combined with a standard attenuation map of the carbon fiber bed determined from transmission scanning. The combined attenuation map was forward projected using software installed on the scanner to produce an attenuation correction factor sinogram, and image reconstruction was repeated with attenuation correction applied. Corrections were also applied for randoms, dead time, normalization, sensitivity, and decay.

Processing of PET Data

Using SPM8 (Wellcome Trust Institute for Neurology, UCL, UK), the MR image of each subject was registered rigidly to a colony-specific structural template produced during previous studies (4). This provided consistent alignment of the MR scans for intra-subject manual rigid registration of PET to MR, which for each PET scan used the mean PET image across all frames. The realigned MR was non-rigidly registered (affine and non-linear) to the structural template using ANTS (5), and this transformation was also applied to the mean PET image co-registered to the realigned MR. Regions of interest (ROI) corresponding to the amygdala were manually defined on the structural template according to the Paxinos et al (2011) marmoset atlas (6), then averaged across hemispheres, manually edited and mirrored about the mid-line to provide symmetric left and right ROIs. The mean PET value (kBq/ml) was normalized by the corresponding mean PET value in a cerebellum ROI to produce standardized uptake value ratio (SUVRc) values. Normalization by the cerebellum signal was designed to minimize the confounding influence of inter-scan differences in tracer availability, plasma glucose concentration, the effect of anesthesia on cerebral blood flow and metabolism, and basal cerebral glucose metabolism. For each scan, an SUVRc map was created for voxel-wise analysis by dividing the mean PET image by the cerebellum ROI value.

For voxel-wise analysis, SUVRc from animals receiving a lesion in the left hemisphere were flipped about the midline such that for all images the intact side of the brain appears on the left. To mitigate against residual registration error and increase data normality, each SUVRc image was smoothed using a Gaussian kernel of $1mm^3$. The kernel was locally

adapted to include only those voxels within a brain mask to avoid contamination from extra-cerebral tissue signal.

Behavioral Analysis

In contrast to the wide array of behavioral responses displayed by a marmoset to a rubber snake when tested in their home cage environment (7), the behavioral repertoire observed in the relatively confined space of the carrying box in the test apparatus was limited. In particular, the animals stayed relatively immobile during the fear-inducing condition and sometimes also during the safety condition. Although not consistent across animals, rearing was seen periodically in the presence of the snake stimulus but no vocalizations were made. The only behavior consistently seen in all animals was that they remained as far away as possible from the location of the snake. Therefore, the floor of the carrying box was divided into a near half and a far half and the duration of time spent in each location was compared in the fear and safety conditions.

It appeared that in the safety condition, in the absence of the snake, marmosets tended to prefer to sit in the ‘near’ position rather than distribute their time evenly across ‘near’ and ‘far’ conditions. Speculatively, the near position was close to the light, open space within the chamber (see Figure 1 in main article), which, without the presence of the snake, may have made it a more attractive place in which to sit.

Cardiovascular Analysis

Blood pressure (BP) data was transmitted by an implanted telemetry probe to a receiver for analysis using Spike2 (Version 7.01, CED) as described previously (8). Outliers and recording failures were removed (values above 200mmHg, below 0mmHg or other abnormal spikes). Data collection was reliable overall, however gaps of less than 0.4s were replaced by cubic spline interpolation and gaps of more than 0.4s were treated as missing values. Systolic BP and diastolic BP events were extracted as local maxima and minima for each cardiac cycle.

Systolic BP at individual time-points were binned into 1s intervals and then calculated as an average over each 1s bin.

Histological Preparation

All marmosets were euthanized with Dolethal (1 ml of a 200 mg/ml solution, pentobarbital sodium, i.p.; Merial Animal Health, Essex, U.K.). Animals were then perfused transcardially with 500 ml of 0.1 M PBS (pH 7.4), followed by 500 ml of 0.4% formaldehyde-buffered solution, washed through over 10 min. The entire brain was removed and placed in fixative solution overnight before being transferred to a 30% sucrose solution in 0.01 M PBS for a minimum of 48h. The brain was then sectioned using microtome into 60µm thick slices. Each brain section was mounted on a slide and stained with cresyl blue.

Histological Analysis

For verification of lesions, coronal sections were viewed under a Leitz DMRD microscope (Leica Microsystems, Wetzler, Germany), and lesioned areas were defined by the presence of major neuronal loss, often with marked gliosis. For each animal, areas with cell loss were schematized onto drawings of standard marmoset coronal sections, and composite diagrams were then made to illustrate the extent of overlap between lesions (Figure 2-B,-C).

Statistical Analysis

For all parametric analyses, i.e. factorial ANOVA, Kolmogorov-Smirnov test was used to test the normality assumption, and Levene's test was used to examine the homogeneity of variance. The assumptions were satisfied unless otherwise noted.

Supplemental Results

Cardiovascular Response of the Three Telemetry Implanted Animals (Control Group) Was Greater During the Fear Condition Than the Safety Condition

Mean systolic BP during each of the four phases (Snake 1, Darkness 1, Snake 2, Darkness 2) in the fear condition (mean of the first exposure and the replication) was compared to the equivalent time periods in the safety condition. The BP was significantly increased during the fear condition in comparison to the safety condition [three-way ANOVA: Condition (Fear vs Safety) x Phase (Snake vs Dark) x Order (1st half vs 2nd half): Main effect of Condition $F(1,2)=89.258$, $p=0.011$; Main effect of Order $F(1,2)=220.887$, $p=0.004$; No main effect of Phase, No significant interaction was found]. *Posthoc* comparison of the fear and safety conditions for each of the phases separately revealed a significant difference during the Snake 1, Darkness 1, and Snake 2, and a trend level difference during the Darkness 2 [One-way ANOVA: $F(1,2)=852.259$, $p=0.001$; $F(1,2)=38.248$, $p=0.025$; $F(1,2)=50.270$, $p=0.019$; $F(1,2)=15.255$, $p=0.060$] (Figure 3-C).

An additional three-way factorial ANOVA in which the two Fear conditions were analysed independently [Condition (1st Fear vs Safety vs 2nd Fear) x Order (1st half vs 2nd half of the session) x Phase (Snake vs Darkness)] revealed Main effect of Condition $F(2,4)=89.240$, $p<0.001$, and Main effect of Order $F(1,2)=175.750$, $p=0.006$ but no Condition x Order x Phase interaction $F(2,4)=2.586$, $p=0.190$. *Posthoc* pairwise comparison revealed that there was a decline in BP scores between the 1st and 2nd fear conditions [mean difference: 10.0±1.1SE, $p=0.011$] suggesting habituation between Fear 1 and Fear 2. Importantly, however, both fear conditions were significantly and independently different from the mean BP score of the safety session [1st Fear vs Safety: mean difference 35.6 ±3.7SE, $p=0.010$; 2nd Fear vs Safety: mean difference 25.6 ±2.8SE, $p=0.012$].

No Behavioral Difference Was Found Between the Lesion Groups and the Telemetry Control Group

When the behavioral pattern in response to the fear or safety conditions was compared between the two lesion groups (antOFC n=7, vlPFC n=4) and the telemetry control group (n=3), no significant group difference was found [three-way ANOVA: Condition (Fear vs Safety) x Distance (Near vs Far) x Group (antOFC vs vlPFC vs Telemetry control): No significant interaction Condition x Distance x Group $F(2,11)=1.658$, $p=0.235$].

No Difference in FDG Uptake in the Intact Hemisphere Was Found Between Left- and Right-Unilaterally Lesioned Animals

The FDG uptake in response to the fear-inducing condition (averaged across Fear 1 and 2) proportional to the safety condition were compared between the animals that had the unilateral lesion in the left or right hemisphere. For the antOFC group (Left=4, Right=3), no significant difference was found either in the '$p<0.001$ insula-amygdala cluster' [one-way ANOVA (Left vs Right): $F<1$; Amygdala cluster $F<1$; Insula cluster $F<1$] (Supplemental Figure S1-A-i). The vlPFC group had three right-hemisphere lesioned animals but only one left-hemisphere lesioned animal. Calculation of the 95% confidence limits for the group of three right-hemisphere lesioned animals showed that the left-hemisphere lesioned animal fell within the range in the '$p<0.001$ insula-amygdala cluster' [Left animal: 5.16%; Right animals: 11.61% ± 7.67%; Amygdala cluster L: 6.82%; R: 12.26% ± 11.29%; Insula cluster L: 4.97%; R: 11.58% ± 7.95%], suggesting that the left-hemisphere lesioned animal was not differentiable from the right-hemisphere lesioned animals (Supplemental Figure S1-B-i).

No Difference in Behavior Was Found Between Left- and Right-Unilaterally Lesioned Animals

Similarly, the behavioral response (distance from the snake) between the left- and right-lesioned animals for the antOFC group showed no effect of hemisphere [three-way factorial ANOVA: Lesion side (Left vs Right) x Condition (Fear vs Safety) x Distance (Near vs Far): No

significant interaction Lesion side x Condition x Distance: $F<1$] (Supplemental Figure S1-A-ii). For the vlPFC group, the left-hemisphere lesioned animal fell within the range of 95% Confidence Interval for the group of three right-hemisphere lesioned animals during the fear-inducing condition [Near: L: 24.25%; R: 33.57% ± 41.48%] (Supplemental Figure S1-B-ii). Note: The 95% confidence limits for the 3 right-lesioned animals in the vlPFC group during 'snake presentation' are large because one of the 3 animals had a tendency to move about during the dark period and freeze as soon as the light came back on and the snake could be seen. In three out of the four snake presentations this animal stopped moving on the near-far border but just within the near sector so their 'far' scores are much lower than the other two animals.

No Difference in Behaviour or FDG Uptake Was Found Between the First and Second Fear Exposures

There were no significant differences in the behavioural response (distance from the snake) between the two conditions (Supplemental Figure S2 i: antOFC group, ii: vlPFC group). Comparison of the FDG uptake of the significant insula-amygdala cluster ($p<0.001$) in the intact hemisphere for the first exposure to the fear-inducing stimuli (Fear 1) and the replication session (Fear 2) revealed no significant differences (Supplemental Figure S3, left upper figure).

No Difference in FDG Uptake Or Behaviour Was Found Between Males and Females

Male and females were compared for the percentage change in the FDG uptake values from the safety to the fear condition in the intact side. No difference was found in any of the clusters: the $p<0.001$ insula-amygdala cluster (mean: male 12.5%±2.0%, female 14.6%±3.0%) [one-way ANOVA: $F<1$], the amygdala cluster (mean: male 9.3%±3.9%, female 14.7%±4.1%) [$F<1$], the insular cluster (mean: male 13.0%±2.0%, female 14.6%±3.0%) [$F<1$]. No difference between male and female was found in the behavioral response [three-way factorial ANOVA: Sex (Male vs Female) x Condition (Fear vs Safety) x Distance (Near vs Far): Main Effect of

Sex $F<1$; Condition x Sex $F<1$; Distance x Sex $F(1,9)=1.069$, $p=0.328$; Condition x Distance x Sex $F<1$].

An Additional Bootstrap Procedure Confirmed that the Decreased FDG Uptake in the Intact Hemisphere in the Safety Compared To Fear Condition Was Not Seen in the vlPFC or antOFC Lesioned Hemisphere

To test the robustness of the findings we performed a bootstrap procedure. For each animal, mean values of SUVR were extracted from the insula-amygdala ($p<0.001$) cluster on the intact and lesioned sides. The mean values for the safety condition were subtracted from the mean fear scan values. Four separate bootstrap procedures were followed, each with 1,000 samples. For the intact hemisphere, resampling was performed from all animals. On the lesioned side, samples were repeated from a) all animals, b) only antOFC-lesioned animals, c) only vlPFC-lesioned animals. Percentile confidence intervals of 95% were calculated from each sample. As can be seen in Supplemental Figure S4, the intact hemisphere of all animals showed differential activation in fear compared to safety conditions but there was no such differential activation on the lesioned side, irrespective of lesion site. Furthermore, there was no evidence that the response to fear vs. safety on the lesioned side varied depending on whether the lesion targeted antOFC or vlPFC.

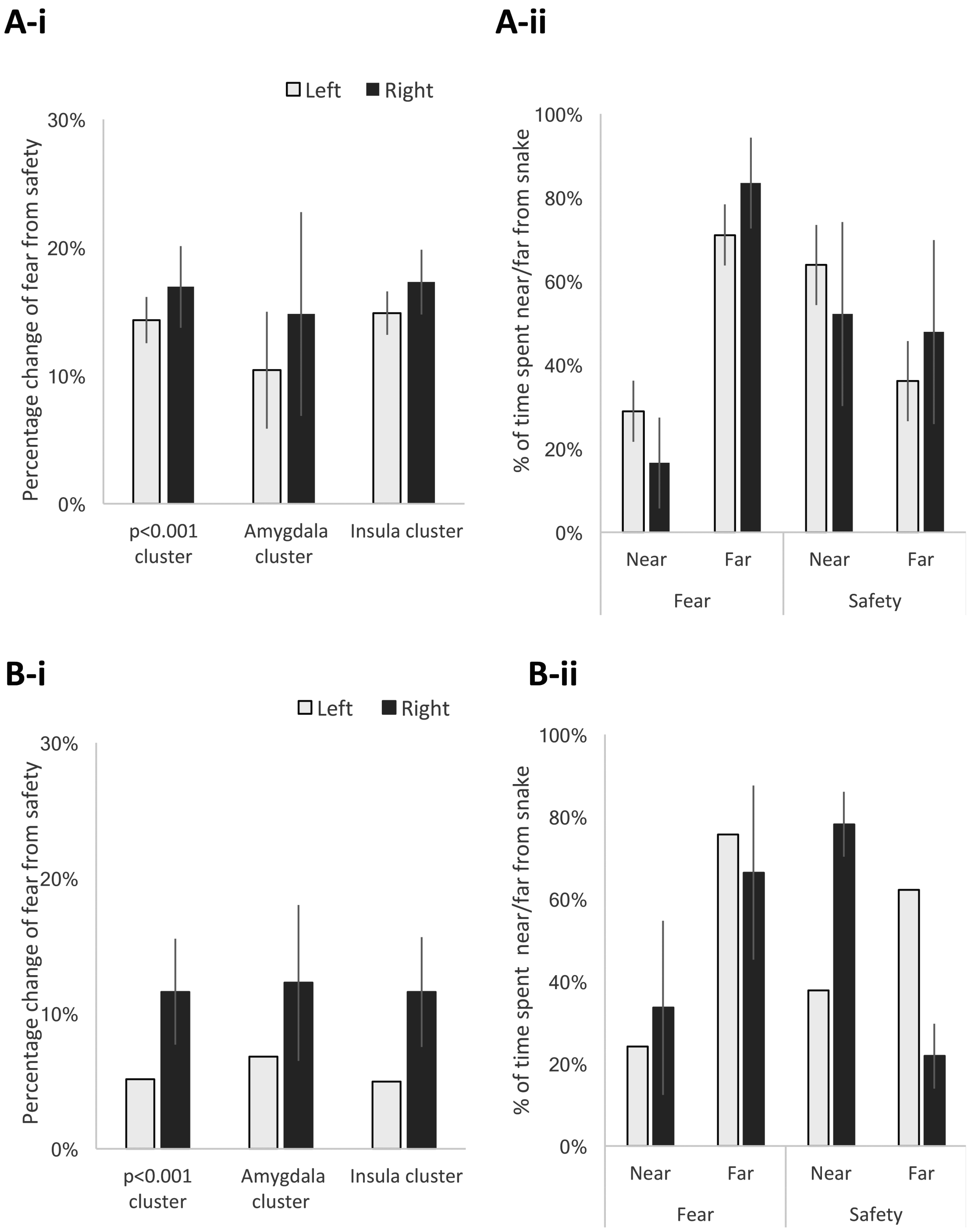

Supplemental Figure S1. Comparison of the FDG uptake in response to the fear-inducing condition (averaged across Fear 1 and 2) proportional to the safety condition of the animals that had the unilateral lesion in the left or right hemisphere for the antOFC group (A-i) (Left=4, Right=3) and vlPFC group (B-i) (Left=1, Right=3). Comparison of the behavioral response (distance from the snake) between the left- and right-lesioned animals for the antOFC group (A-ii) and vlPFC group (B-ii). No significant difference was found between the left and right lesioned animals in any of the measures.

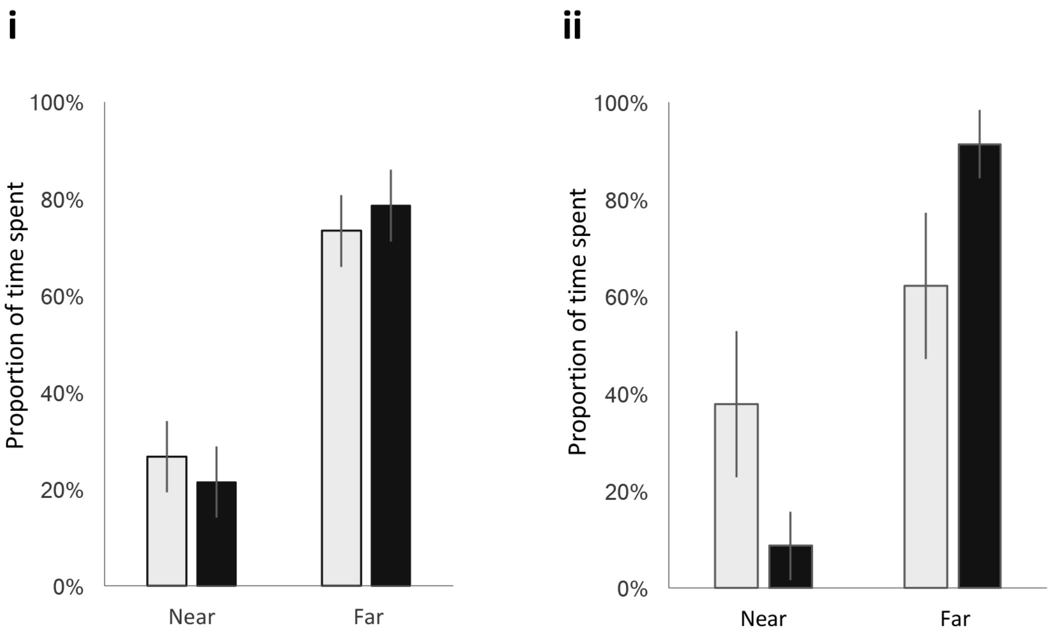

Supplemental Figure S2. Comparison of the behavioural response (distance from the snake) during the first exposure to the fear-inducing stimuli (Fear 1; grey bars) and the replication session (Fear 2, black bars) for the antOFC group (i) and the vlPFC group (ii). There was no statistically significant difference between the first exposure and replication session. Error bars indicate standard errors.

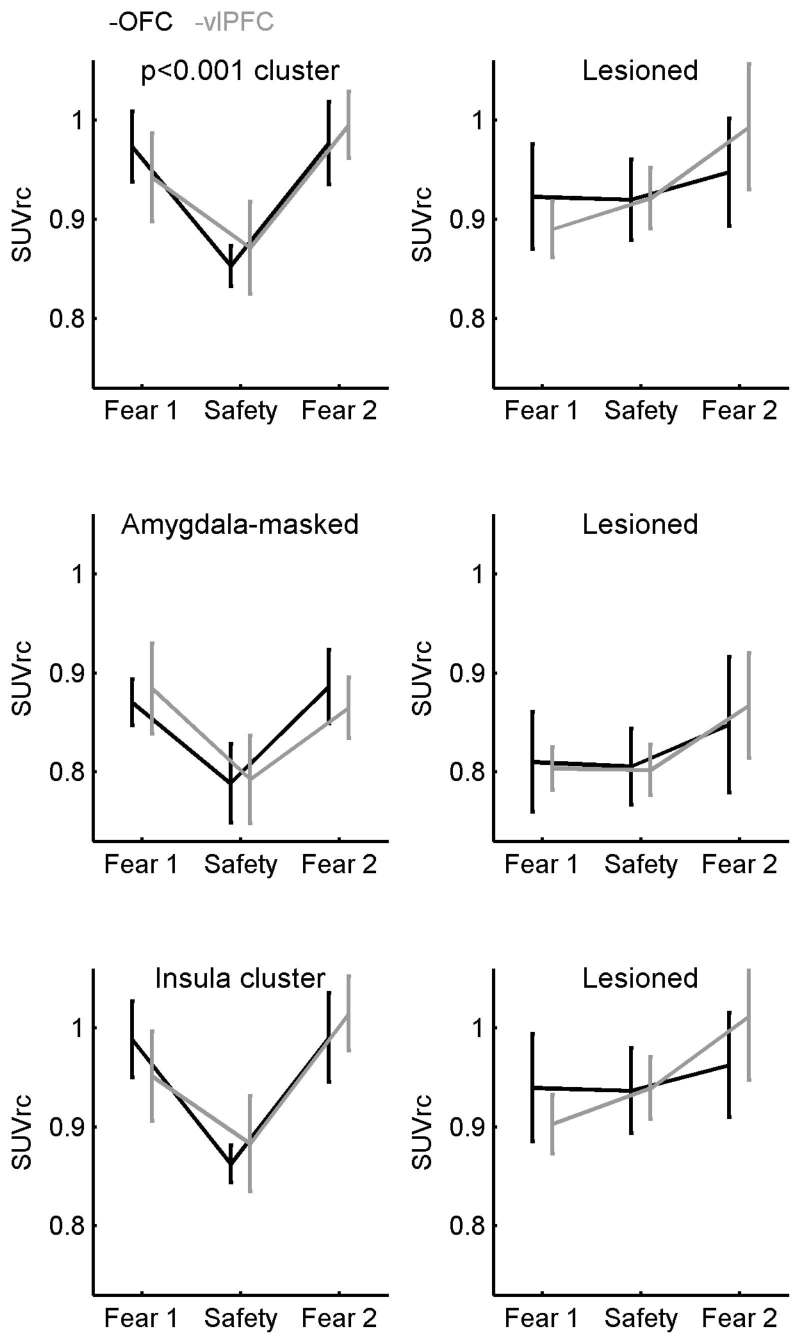

Supplemental Figure S3. Adjusted means of the FDG uptake scores in the intact (left-hand side) and lesioned hemispheres (right-hand side) for the significant insula-amygdala cluster [p<0.001] (upper figures), the same cluster masked by the amygdala ROI (middle figures) and the remaining insula cluster (lower figures), across the three conditions (first fear-induction; Fear 1, safety, fear replication; Fear 2). Adjustment was performed by fitting a general linear model with effects of Subject and Condition. Data are shown with individual subject effects removed and replaced with a mean subject effect. Error bars show 95% confidence intervals.

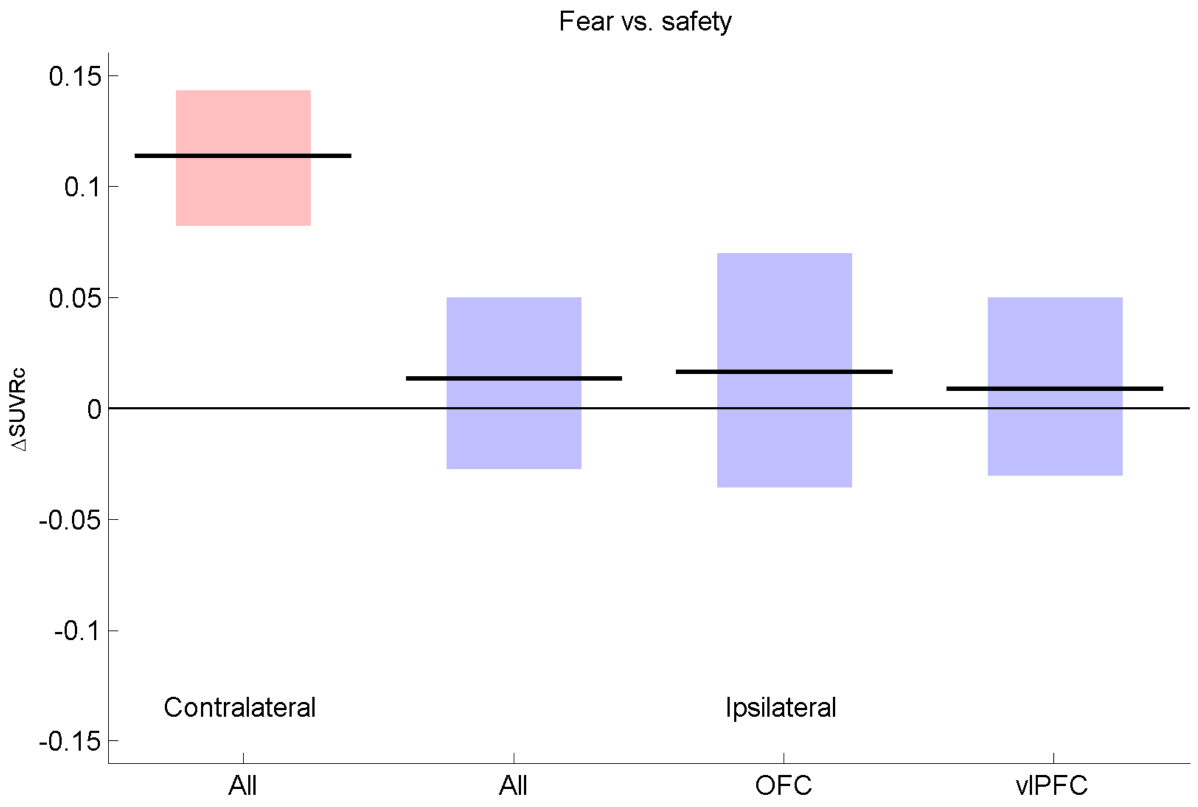

Supplemental Figure S4. Percentile 95% confidence intervals. In the contralateral intact hemisphere the difference in FDG uptake during fear compared to safety was significantly different from ‘0’. In contrast, all ipsilateral lesioned hemispheres (All), antOFC lesioned only (OFC) or vlPFC lesioned only (vlPFC) did not differ from ‘0’ and were significantly lower than the intact hemisphere.

Supplemental References

1. Roberts AC, Tomic DL, Parkinson CH, Roeling TA, Cutter DJ, Robbins TW, Everitt BJ (2007): Forebrain connectivity of the prefrontal cortex in the marmoset monkey (Callithrix jacchus): An anterograde and retrograde tract-tracing study. *J Comp Neurol* 502: Wiley Online Library86–112.

2. Defrise M, Kinahan PE, Townsend DW, Michel C, Sibomana M, Newport DF (1997): Exact and approximate rebinning algorithms for 3-D PET data. *IEEE Trans Med Imaging* 16: 145–158.

3. Hudson HM, Larkin RS (1994): Accelerated image reconstruction using ordered subsets of projection data. *IEEE Trans Med Imaging* 13: 601–609.

4. Mikheenko Y, Shiba Y, Sawiak SJS, Braesicke K, Cockcroft G, Clarke HF, Roberts AC (2014): Serotonergic, brain volume and attentional correlates of trait anxiety in primates. *Neuropsychopharmacology*Nature Publishing Group1–10.

5. Avants BB, Epstein CL, Grossman M, Gee JC (2008): Symmetric diffeomorphic image registration with cross-correlation: Evaluating automated labeling of elderly and neurodegenerative brain. *Med Image Anal* 12: 26–41.

6. Paxinos G, Watson C, Petrides M, Rosa M, Tokuno H (2011): The Marmoset Brain in Stereotaxic Coordinates. New.San Diego, USA: Elsevier Science Publishing Co Inc.

7. Shiba Y, Santangelo AM, Braesicke K, Agustín-Pavón C, Cockcroft G, Haggard M, Roberts AC (2014): Individual differences in behavioral and cardiovascular reactivity to emotive stimuli and their relationship to cognitive flexibility in a primate model of trait anxiety. *Front Behav Neurosci* 8: 1–14.

8. Braesicke K, Parkinson JA, Reekie Y, Man M-S, Hopewell L, Pears A, *et al.* (2005): Autonomic arousal in an appetitive context in primates: a behavioural and neural analysis. *Eur J Neurosci* 21: 1733–40.
